# The relationship between working in the “gig” economy and perceived subjective well-being in Western Balkan countries

**DOI:** 10.3389/fpsyg.2023.1180532

**Published:** 2023-06-12

**Authors:** Miloš Vučeković, Goran Avlijaš, Mirjana Radović Marković, Dejan Radulović, Arsen Dragojević, Dušan Marković

**Affiliations:** ^1^Faculty of Business, Singidunum University, Belgrade, Serbia; ^2^Faculty of Economics and Engineering Management, Business Academy University, Novi Sad, Serbia; ^3^Business Academy, Novi Sad, Serbia; ^4^Ipsos Strategic Marketing, Belgrade, Serbia; ^5^Belgrade Business School, Belgrade, Serbia

**Keywords:** freelancers, remote work, subjective well-being, gig economy, Western Balkan countries

## Abstract

The combination of accelerated digitalization and the recent COVID-19 crisis has increased the number of remote workers worldwide to unimaginable proportions. Among the large number of remote workers that execute their projects from home, there is a significant number of permanently self-employed remote workers, usually referred to as freelancers. Despite the importance of this kind of business activity for modern project management society, perceived drivers of freelancing are still unknown. The goal of this paper was to shed some light on the general subjective well-being of freelancing activity and investigate differences concerning gender, age, and education. The study was performed in late 2020 and included 471 freelancers from Serbia, Bosnia and Herzegovina, Macedonia, and Montenegro that participated in an online questionnaire evaluating their subjective well-being while participating in the “gig” economy. Factor analysis was used as a primary statistical method and two major groups were identified: (1) Impact of working from home on a freelancer’s personal life and health and (2) Fulfillment of expectations in the economic and professional sense. Gender was found not to be significant for overall work satisfaction. However, older freelancers proved to be more satisfied with the fulfillment of economic and professional expectations, which correlate with years of professional experience. Another conclusion is that more educated freelancers are generally less satisfied with both groups of drivers - fulfillment of personal life and professional expectations. Understanding how the combination of occupations, technological infrastructure, and demographic characteristics in the region has affected the well-being of freelancers may help policymakers and organization owners, as well as future entrepreneurs, better prepare for this model of work in the future. It also increases the possibility of exploring individual dimensions of wellbeing useful for targeting interventions at the level of each country separately. In line with this, the present study contributes to the existing body of knowledge and the impact of hybrid models of work on the subjective well-being of workers in the “gig” economy.

## 1. Introduction

European economies have faced a significant increase in self-employed individuals over the last two decades. One of the most significant subgroups among these self-employed individuals is the freelancer group (Van den Born and Van Witteloostuijn, 2014). Freelancers are considered professional individuals contracted by companies and others to whom they sell their knowledge or services, performing at their own risk with temporary work arrangements (Kitching and Smallbone, 2012; Bögenhold and Klinglmair, 2016).

The latest report from the Online Labor Index (OLI) that provides information on the current state of the freelance (gig) economy ranks Western Balkans Countries (WBC) relatively high by the number of freelancers, putting Serbia in 12th, Bosnia and Hercegovina in 47th, North Macedonia in 70th and Montenegro in 110th place on the global scale (Lehdonvirta et al., 2020).

Satisfaction with personal life and subjective well-being (SWB) indicates how individuals feel and perceive their lives, with many studies associating cognitive evaluations of people’s lives with various individual characteristics of responders (Meager, 2015; Van Stel and Van der Zwan, 2020). However, the subjective well-being of workers in the gig economy is still a newer topic that needs further research and investigation, expanding on previous findings (Diener et al., 2003; Dolan et al., 2008; Binder and Coad, 2016).

This paper aims to contribute to the increasing body of knowledge by shedding some light on the SWB of freelancers and drivers that contribute to the significant increase of these activities in the WBC region (Serbia, Bosnia and Herzegovina, Montenegro, and North Macedonia).

The paper addressed the research problem using a two-phase analysis to understand the relationship between working in the “gig” economy and perceived subjective well-being. Since SWB accompanies multiple life domains (Erdogan et al., 2012; Binder and Coad, 2013), factor analysis helped measure correlation with each other and decrease the number of survey items to the most relevant factors to use in the following phase. The second phase investigated the differences within the group of freelancers by utilizing gender, age, and education as variables, similar to previous studies (Larsson and Thulin, 2019; Van der Zwan et al., 2020).

The structure of this paper is as follows: The first section introduces the main goals and starting points of this research paper. The second section showcases a review of the existing literature on the “gig” economy, subjective well-being, and drivers of freelancing activity. The third section provides information on research methodology, which includes a description of the data and variables used, statistical approach, and data preprocessing. The Results, discussion, and final remarks are in the last two sections.

## 2. Literature review

There are many definitions of what “gig economy” means, some describing it as an exchange of labor for money between different parties over online platforms that enable matchmaking and payments between them (Broughton et al., 2018), while others have a broader definition that covers all hybrid work of highly specialized tasks engaged in over digital platforms (Abraham et al., 2018).

Freelancing is usually associated with the specific relationship between a client and a worker that is not permanently working for that client (Wood et al., 2018), sometimes regarded as a new type of entrepreneurship, especially in emerging markets (Akhmetshin et al., 2018). The main characteristics of freelancing are peculiar, project-based, low waged, and infrequent work engagements (Osnowitz, 2010; Cohen, 2015; Aletdinova, 2016; Gandini, 2016; Lo Presti et al., 2018; Radović-Marković et al., 2021c; Worth and Karaagac, 2021), making a clear shift from the Fordist ideal of stable employment to a more neoliberal narrative of free engagement in the unregulated markets (Popiel, 2017; Bologna, 2018). Uberization of work, the new term defining peculiar types of work over digital platforms, also made it possible to recruit labor in new ways and at a larger scale (Davis and Sinha, 2021).

Furthermore, an increasing number of people work in project-based non-traditional environments without a clearly defined organizational structure, shared workspaces, and long-term engagements, which fundamentally changes the relationship dynamics in these organizations between workers and employers (Ashford et al., 2018). They generally work independently and without supervision. Accordingly, this type of autonomy in the “gig” economy creates a specific relationship between workers and organizations, enabling the execution of tasks without active participation in the employer’s business organization.

### 2.1. Drivers and challenges of working in the “gig” economy

Virtual project teams are gradually becoming a norm as more and more companies move to the remote-first or async strategies that go beyond outsourcing and employing freelancers on company projects. Of course, leading this kind of project where there is at least one remote team member may pose different challenges than when all the team members are collocated. There is also a notable rise in the popularity of working as a freelance project manager (Project Management Institute, 2017), which may add additional strain to team interactions.

In a survey conducted among professionals working remotely, “engaging remote participants” was the most common response to the question of challenges in remote project settings (Pullan and Prokopi, 2016), and the main issue when employing freelancers on projects is enabling the shared understanding between team members, which may be paramount for success in remote scenarios (Kniel and Comi, 2021). In a book that covered leading remote teams, the author argued that the novel coronavirus changed the perspectives of working remotely, making almost everyone a remote contributor to the organization, thus requiring new ways of managing and leading the workforce during the pandemic (Burkus, 2021).

When comparing aspects of remote work to the “Agile Manifesto” principles, which emphasize face-to-face conversations and team meetings, the authors of another study on a similar topic conclude that there are significant differences between collocated and remote teams (Deshpande et al., 2016). Therefore, there may be a need to have a decision model for choosing the different PM methodologies for each specific case (Thesing et al., 2021) or even look for a whole new approach to project management on an organizational level (Picciotto, 2020). In addition, multiple software solutions are needed for successfully managing these kinds of projects, especially for task management, teleconferencing, and distance training (Borissova et al., 2020). Despite these findings, the value creation of remote teams seems to be on par with that of co-located teams, which was battle-tested during the COVID-19 abrupt shift to remote work by major international companies (Comella-Dorda et al., 2020).

#### 2.1.1. The current research on working from home, freelancing, and “gig” economy in WBC region

In a recent study on the social and economic advantages and limitations of working from home in the region of Western Balkans it was showcased that the most participants would recommend working in this manner, with some still hesitant to do so (Đukanović et al., 2022). Furthermore, the study acknowledged the benefits of working from home and found that the main motivators for respondents starting home-based businesses were better work-life balance, a higher possibility of earning a better income, and a good business idea. Similarly, a study conducted among participants of the “gig” economy in the WBC region shows that good income and flexibility of time schedules are the most important motivators for people engaged in this way (Borislav et al., 2022), with freelancing cutting across all ages and education levels, and giving much greater independence and autonomy to the workers, thus enabling higher satisfaction in working from home as mentioned previously (Đukanović et al., 2022).

In addition, new forms of employment that include platform work or working remotely by utilizing online marketplaces are gaining more popularity because of the very high unemployment rates in the WBC region and better opportunities to earn a decent income (European Training Foundation, 2022).

#### 2.1.2. Challenges of freelancing

One number of analyzes showed that there is a trend of deteriorating working conditions and worker rights in the “gig” economy, directly comparing the present situation in the markets with the working conditions at the beginning of the past century (Flanagan, 2019). Furthermore, there is a high demand for government intervention in enabling independent arbitrage in case of a dispute or request for personal records ownership, but the triangular relationship of gig work between the platforms, clients, and workers complicates the task of labor regulation, as there are multiple agreements between parties in place (Stewart and Stanford, 2017).

The Terms of Service usually define the relationship between intermediaries and clients where the risk of providing necessary tools and equipment is on the worker, which enhances vulnerability and instability for them (Sutherland et al., 2020). Also, the same agreements enable platforms to enforce disciplinary measures and generally supervise the work conducted by the workers (Jarrahi and Sutherland, 2019). Technology-enabled online work platforms rely heavily on instant feedback and rating of the worker’s performance (De Stefano, 2015). Furthermore, there is an evident attempt by them to impose restrictions on the workers and restrict employment agreements between the parties, contacts by third parties about future work, and accepting any offers that come outside their system.

On the other hand, the agreement between the intermediaries and the clients usually diminishes any liability for platforms (Purcell and Garcia, 2021). The authors of the previously mentioned research (Stewart and Stanford, 2017) suggest many options for extending the regulation, but some doubt that regulating the “gig” economy is that easy or even, in some cases, possible (Collier et al., 2017). Despite this, the same research shows the disadvantages of this kind of engagement, including collapsing traditional relations between employees and employers, the ever-present deprivation of workers’ rights to sick leave, annual leave, compensation after dismissal, and the like.

Furthermore, freelancing takes a toll on workers by introducing precarity, highly intense stress, uncertainty, and alienation into everyday life by blurring the lines between personal and work commitments (Đukanović et al., 2022).

#### 2.1.3. Drivers of freelancing activity

Many studies have been done on the topic of drivers and experiences of workers in the gig economy during recent years, trying to identify and explain the motivators, incentives, and reasons for participation in this type of economy (Allon et al., 2018; Ashford et al., 2018; Bajwa et al., 2018; Berger et al., 2019; Van der Zwan et al., 2020). For example, in one large study on the topic of economic and behavioral drivers of workers in the “gig” economy, the authors concluded that the possibility of higher earnings and the flexibility of choosing working hours are the primary drivers of engaging in this kind of work (Allon et al., 2018), similarly to the previous research findings in the region of Western Balkans (Radović-Marković et al., 2021a,b). Some people also freelance to “survive” until the next full-time opportunity arrives by moving between employment and unemployment, thus painting a different picture of freelancer motivation for joining the “gig” economy (Menger, 2017). On the other side, previous research showed that freelancers provide vast value for companies and employers (Mettler and Williams, 2011; Burke and Cowling, 2015; Aloisi, 2016; Barlage et al., 2019; Burke and Cowling, 2020; Gupta et al., 2020) who are incentivized to engage workers in the “gig” economy for different kinds of projects. Furthermore, there is evident relationship between high level of self-fulfillment and happiness and entrepreneurial activity, as previous studies show (Núñez-Barriopedro et al., 2020; Ravina-Ripoll et al., 2021).

### 2.2. Components of subjective well-being

Throughout history, happiness has been the highest good and the greatest motivation for human action within the experience of the individual making subjective well-being a central point in measuring the quality of life (Campbell, 1976; Diener, 2009). Dimensions for assessing SWB are restricted to life satisfaction domain (cognitive measure) in this research paper, and include a global subjective evaluation of the respondent’s life (Dolan and Metcalfe, 2012). Furthermore, the experiences of individuals vary across different domains of life, and they may be valued differently among the respondents (Ryff and Heidrich, 1997).

The literature also shows that evaluative questions are usually used for this purpose (Kahneman and Krueger, 2006), with a standard five-item response scaling system (Diener et al., 1985) and a Life Satisfaction Scale (SWLS) (Diener, 2000). Also, it is common to have evaluative measures in nationally conducted studies on SWB (Kapteyn et al., 2015), with single-item and multi-item measuring scales used in previous research studies on this topic (Andrews and Crandall, 1976; Haring et al., 1984; Okun et al., 1984; Witter et al., 1985; Okun and Stock, 1987; Ferrans and Powers, 1992; Ellingson and Conn, 2000).

#### 2.2.1. Gender and subjective well-being of freelancers

There is a growing trend among women to become solo entrepreneurs to fulfill other life commitments while attaining a better work-life balance (Bögenhold and Klinglmair, 2015). Furthermore, some research shows the significant success of solo entrepreneurship as a strategy to cope with family obligations and take care of the children while earning an income (Duberley and Carrigan, 2013). Regarding the pay gap, research that included freelancers from Ukraine, India, Pakistan, the United States, and Bangladesh concluded that female freelancers are underpaid in almost all categories and usually undervalue themselves when bidding for jobs (Dubey et al., 2017). In addition, other research has shown different pricing strategies that participants in the gig economy have, which directly impact how much they earn by working in this manner (Foong and Gerber, 2021). Lastly, one study conducted during the COVID-19 pandemic in 2020 showcased how women freelancers carry an additional burden of household obligations, thus sacrificing work time to provide caregiving to family members or finish other in-house chores (Dunn et al., 2021).

#### 2.2.2. Age and subjective well-being of freelancers

Better access to flexible work arrangements may become crucial for the older population to be engaged in the labor market but may provide fewer benefits for younger participants in the “gig” economy (Cory, 2012). Also, engaging in this type of economy can be beneficial for semi-retired workers who want to earn additional funds alongside their pension, but their position in it may not be ideal in a market that only compensates workers based on their achieved productivity (Cook et al., 2019). There is a clear positive impact of marital status and subjective well-being as well as between being religions and fulfillment of expectations (Ravina-Ripoll et al., 2022).

Furthermore, previous research done in the United Kingdom on the topic of employment of people over 50 years old showcased the impact the “gig” economy has on enabling flexible work arrangements for elderly workers engaged in portfolio jobs, freelancing, or consulting (Platman, 2004), but various occupational health implications should also be considered (Tran and Sokas, 2017) before any conclusion on the benefits of this kind of work for elderly people can be made.

#### 2.2.3. Education and subjective well-being of freelancers

On the topic of education, there is a clear trend toward lifelong learning and on-demand certification that is becoming very important in the “gig” economy, as workers need to attain new skills quickly and showcase their proficiency to stay relevant in this type of economy (Wheelahan and Moodie, 2022). Furthermore, investing in self-education may be a great strategy to stay afloat in the labor markets, especially in the era of shifting to more competency-based curriculum. In a broader sense, the complexity of understanding the needs of labor markets may blur the lines between formal and informal education programs, especially when considering that engagement in this kind of economy includes not only platform work but other types of casual work arrangements as well (Forum, 2020).

Because of such activities, often employees in the “gig” economy fail to define their career path, sometimes having too many options in choosing the next job they will work on, which ultimately leads to demotivation and dissatisfaction and shifts the “radical responsibility” of short-term or long-term economic survival to the worker. Also, workers in the “gig economy” cannot expect training from employers, so they must complete the relevant training and education in their own time to perform specific assignments. Specialization has proven not to be a good long-term strategy for workers in this economy, as market demands frequent change. Consequently, participants in this economy must be committed to learning throughout their lives, i.e., to renew their knowledge and move between different areas of expertise.

Considering the findings showcased above, we propose hypothesis as follow:

*H1*: *Working as a freelancer impacts subjective well-being in the domain of life satisfaction.*

*H2*: *Subjective levels of well-being in the domain of life satisfaction can be highly affected by a freelancer’s gender, age, and education.*

## 3. Research methodology

### 3.1. Survey instrument and variables

The online survey was conducted in 2020 during the COVID-19 pandemic while workers practiced remote work in a new setting and included 13 questions on freelancers subjective-well-being and experiences working in the “gig” economy. Our research focused on investigating the subjective well-being of 989 respondents including 370 respondents from Serbia, 197 from Montenegro, 221 from North Macedonia, and 201 from Bosnia and Herzegovina.

Afterwards, we excluded the responses with missing data and the ones that stated having permanent work contracts from the data sample to examine satisfaction among freelancers only and to maintain data integrity. Therefore, the final data sample included 471 freelancers whose SWB was analyzed and measured.

The questionnaire was made based on the literature review and commonly found topics on this subject. The provided answers to the asked questions were given to the respondents in a form of five-point Likert-type scale measuring SWB from 1 (Completely disagrees) to 5 (Completely Agrees).

**Figure 1:**
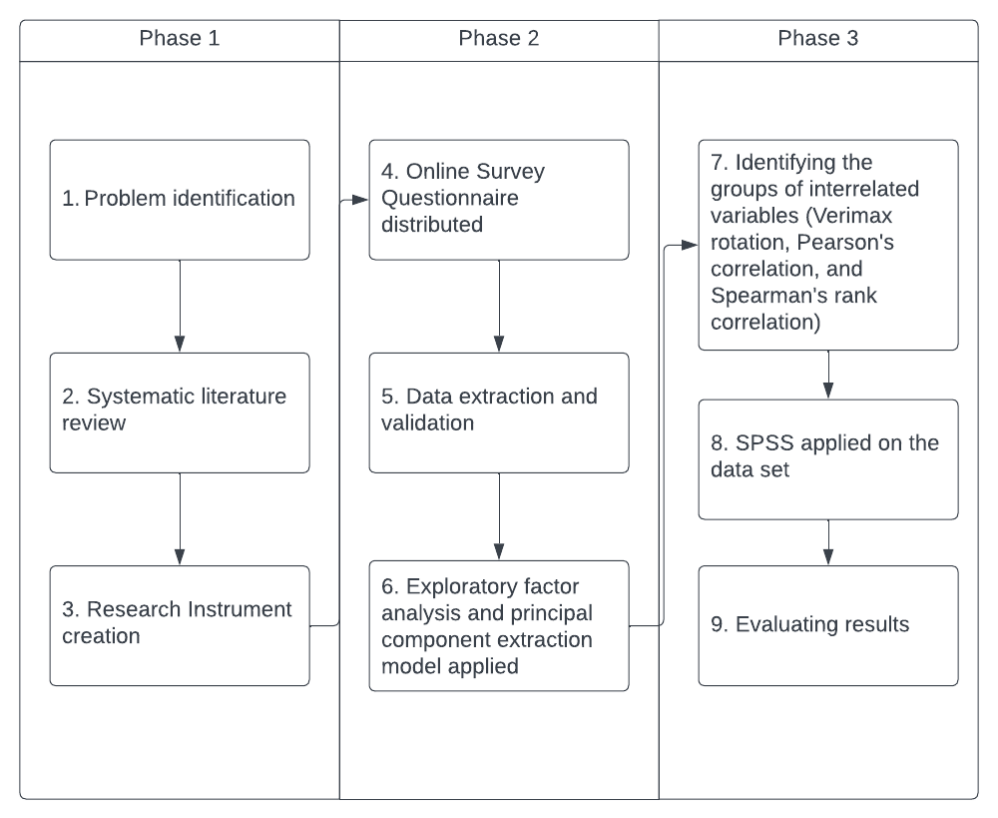
Research approach.

Because of the multidimensional nature of the research model, exploratory factor analysis (Gorsuch, 1988; Abdi and Williams, 2010; Williams et al., 2010) and the principal component extraction model were used to reduce the number of variables to common factors and help us understand the underlying concepts. Furthermore, the varimax rotation (Jackson, 2005; Corner, 2009), Pearson’s correlation (Benesty et al., 2009), and Spearman’s rank correlation (Gauthier, 2001) are used on the dataset to identify the groups of interrelated variables and investigate how they relate, enabling us to separate factors that are orthogonal to each other, ie. they have 0 correlation which allows them to be better and easier to demarcate. The SPSS software was used to complete the statistical analysis of the attained data sets (Figure 1).

## 4. Key findings

The Cronbach’s alpha coefficient (Ercan et al., 2007; NOKS, 2023) validated the reliability of the instrument (0,830). Item total statistics confirmed that discarding any questions would not impact the scale. Exploratory factor analysis with varimax rotation supports the structure of the questionnaire (Gorsuch, 1988; Jackson, 2005; Corner, 2009; Williams et al., 2010). The first-factor solution shows three factors with a characteristic root greater than 1. But as the third factor consists of only two items and as the scree plot (Macrosson, 1999) also indicated a two-factor solution, we decided to repeat the analysis and limit the number of factors to two (Table 1).

**Table 1:** Data sample and variables used for the research.

Country	Gender		Age			Education			Total
	Male	Female	<26	26–35	>35	High School	*College	University	
Bosnia and Herzegovina	75	54	95	25	9	93	6	30	129
Montenegro	19	26	12	23	10	12	2	31	45
North Macedonia	70	61	36	49	46	52	36	43	131
Serbia	101	65	41	63	62	83	34	49	166
Total	265	206	184	160	127	235	78	153	471

^*^College option equivalent to the NQFS scale definition (Corner, 2009).

The results displayed in Table 2 show the demographic data of the participants from this research by gender, age, and education. The majority of the participants of this study attained a High School degree (235), and the others have either College (78) or University (153) levels of education. Regarding age, most participants are 26 years old or less (184), then the second group is younger adults aged from 26 to 35 years (26–35), and then the adults aged 35 years or more (127).

**Table 2:** Dimensions and main topics found during the literature review.

Author/Year	Dimensions	Main topics
Đukanović et al. (2022)	Material, psychosocial	Working from home, social and economic expectations and satisfaction
Bögenhold and Klinglmair (2015)	Gender, material	Female entreprenership, self-employment, freelancing
Binder and Coad (2016)	Health, social, SWB	Life domains, self-employment, Subjective well-being
Taboroši et al. (2022)	Gender, age, education	Entreprenership, freelancing
Vučeković et al. (2021)	Gender, fulfillment in economic sense	Woman entreprenership, freelancing

The Kaiser-Meyer-Olkin (KMO) test (Dziuban and Shirkey, 1974) indicates that the collected data is suitable for factoring (Table 3). By using exploratory factor analysis with varimax rotation, two factors were singled out. The first factor consists of 7 items, and the second factor consists of 6 items. The first factor describes the impact of remote work on the freelancer’s personal life and health. The second factor represents the fulfillment of expectations in the economic and professional sense (Table 4). The question “Since I work from home, I have more time to spend with friends” displayed the same lower saturation for both factors.

**Table 3:** Total variance explained.

Component	Initial eigenvalues			Extraction sums of squared loadings			Rotation sums of squared loadings		
	Total	% of Variance	Cumulative %	Total	% of Variance	Cumulative %	Total	% of Variance	Cumulative %
1	4.430	34,079	34.079	4.430	34.079	34.079	3.100	23.843	23.843
2	1.410	10.843	44.922	1.410	10.843	44.922	2.740	21.079	44.922
3	1.111	8.547	53.470						
4	0.945	7.266	60.736						
5	0.848	6.521	67.257						
6	0.696	5.354	72.611						
7	0.674	5.184	77.794						
8	0.620	4.767	82.561						
9	0.540	4.151	86.712						
10	0.491	3.779	90,491						
11	0.448	3.449	93.940						
12	0.404	3.110	97.050						
13	0.384	2.950	100.000						

Extraction method: principal component analysis.

**Table 4:** KMO and Bartlett’s test.

Kaiser-Meyer-Olkin measure of sampling adequacy		0.866
Bartlett’s test of sphericity	Approx. Chi-Square	3286.374
	df	78
	Sig.	0.000

Using the results from the rotated component matrix (Table 5), the following two main factors were identified with correlation to the survey questions:

- Factor 1–The impact of working from home on a freelancer’s personal life and health
- Factor 2–Fulfillment of expectations in the economic and professional sense

## 5. Discussion

The results show a high negative impact of working from home on subjective well-being, with respondents acknowledging feeling tense and anxious, having trouble separating work and private life, and neglecting other commitments, as also found in the previous research on this topic (Dubey et al., 2017; Tran and Sokas, 2017; Van der Zwan et al., 2020). There is also a high correlation between the fulfillment of expectations in the economic and professional sense and freelancing, which was also presented previously in the literature review.

Furthermore, their subjective well-being may vary depending on their motives for joining this kind of economy and the source of income that it provides, as previous studies have shown (Huđek et al., 2021). This paper found similar results, showing differences in satisfaction levels based on two main factors.

Regarding gender, the analysis did not find any significant differences by using the t-Test on the independent samples, confirming the findings of a recent study done in the same region on a similar topic (Vučeković et al., 2021; Taboroši et al., 2022). The research on the “gig” economy is still in its infancy compared to the traditional markets, and further research is needed to understand the dynamics of this type of economy and its benefits and drawbacks. Still, there is a discrepancy between the pricing and negotiation techniques among the genders found in similar research during the literature review, which may impact the subjective well-being of the involved participants in this economy (Chung and Hahn, 2021).

There is also a clear correlation between respondents’ age and the fulfillment of expectations in a monetary and professional sense (Table 6), as many freelancers are domain experts and professionals offering their services in an additional capacity (Platman, 2004). Previous studies covered in the literature review show the significant advantage of professional workers in the “gig” economy with many years of work experience against the younger population, but not the case in the transportation and delivery industry (Younger, n.d.).

**Table 5:** Rotated component matrix.

	Component	
	1	2
Since working from home, I feel more tense and anxious	0.733	0.224
I have trouble separating my work from my private life	0.725	0.163
Since I’ve been working from home, I’ve paid less attention to my family or home.	0.713	0.277
Working from home had a negative impact on the quantity and quality of sleep.	0.694	0.224
Since I started my own business, I have much less time for myself and my hobbies	0.611	0.270
I would like to have more contact with other people during work	0.499	−0.024
Since I work from home, I have more time to spend with friends.	0.355	0.319
My current income is sufficient to cover all my basic needs.	−0.020	0.785
I am confident in the stability and future success of the work I am currently doing	0.062	0.780
The work I am currently doing fulfills me.	0.219	0.617
My family and close friends mostly support me in my current job.	0.332	0.558
While working from home, others (family, friends, neighbors) do not disturb me, and I can fully devote myself to work.	0.285	0.512
Working from home does not prevent me from allocating enough time and motivation to engage in physical activities (exercise/sports).	0.233	0.428

Extraction method: principal component analysis. Rotation method: varimax with kaiser normalization. ^a^Rotation converged in 3 iterations.

Between the level of education and both factors negative correlation was also found (Table 7), showcasing that with the increase in the level of education, dissatisfaction grows in domains of subjective well-being as well as in monetary and career fulfillment, very similar to the previous findings (Menger, 2017; Ashford et al., 2018; Sutherland et al., 2020).

**Table 6:** Correlations between variable age and factors.

		Age	Factor 1	Factor 2
Age	Pearson correlation	1	0.024	0.109^*^
	Sig. (2-tailed)		0.602	0.018
	*N*	471	471	471

*Significant correlation at the 0.05 level (2-tailed).

Furthermore, there is a clear benefit for organizations in regard of resilience and business continuity by engaging workers in the „gig “economy, especially when implementing already available frameworks for that purpose (Marković, 2018).

Based on the findings above, we conclude that the first hypothesis “Working in the “gig” economy impacts subjective well-being in the economic, professional, and personal life domains” is confirmed and that the second hypothesis “The subjective well-being levels depends on freelancers’ gender, age, and education” is only partially confirmed with no significant differences when considering gender (Tables 7, 8).

**Table 7:** Correlations between variable education and factors.

			Factor 1	Factor 2	Education
Spearman’s rho	Education	Correlation coefficient	−0.225^**^	−0.097^*^	1.000
		Sig. (2-tailed)	0.000	0.035	
		*N*	471	471	471

*Significant correlation at the 0.05 level (2-tailed). **Significant correlation at the 0.01 level (2-tailed).

**Table 8:** Statistical analysis.

Statistical method	Notes
Cronbach’s alpha coefficient	Validated the reliability of the instrument
The Kaiser-Meyer-Olkin (KMO) test	Checking if the data is suitable for factoring
Exploratory factor analysis with varimax rotation	Separating factors that are orthogonal to each other (they have 0 correlation)
Principal component analysis	Standard extraction model
Pearson’s correlation	Used on age variable because it is a continuous variable
Spearman’s rank correlation	Used on education variable because it is of ordinal type

## 6. Conclusion

The contribution of our research is that it is one of the first attempt to empirically explain the relationship between freelancing and subjective well-being in Western Balkans. In line with this, our study concludes that flexible working conditions, professional and economic fulfillment, and improved work-life balance are the primary motivators for freelancers working in the “gig” economy in selected countries. Furthermore, there is a clear trend regarding heightened subjective well-being scores and freelancing from home compared to workers who provide traditional services from home, especially when engaged in the passion economy.

The analysis of workers in the “gig” economy concerning their subjective well-being reveals that satisfaction levels drop significantly with the education level of the respondents. Also, there seems to be a trend toward higher satisfaction among the older population participating in the gig economy. As for several other domains of SWB, there are differences between highly educated workers performing their domain work and those working on low-skilled tasks, like transportation or delivery. Furthermore, the results did not show any significant differences between genders among freelancers, but the impact of age and education variables is observable.

By reviewing the survey results and showcasing the findings, this paper contributes to the previous body of knowledge on the topic of motivators for freelancing and its impact on SWB of the participants in the “gig” economy in a limited capacity and calls for further research by investigating the impact of self-employment and peculiar work arrangements on the quality of life and other domains of subjective well-being. Furthermore, limiting factors of our research are related to the fact that due to the pandemic, we were able to collect data electronically, i.e., through a web questionnaire and not directly in the field. Additionally, the authors recommend analyzing the differences between freelancers, self-employed individuals, and part-time contingent workers and their perspectives on subjective well-being in future research.

## Data availability statement

The original contributions presented in the study are included in the article/supplementary material, further inquiries can be directed to the corresponding author.

## Ethics statement

Ethical review and approval was not required for the study on human participants in accordance with the local legislation and institutional requirements. The patients/participants provided their written informed consent to participate in this study.

## Author contributions

MV and GA prepared the research concept and literature review, and gave their take on the results. MM defined the methodology and questionnaire and provided final conclusions. DR reviewed and provided perspective on the legal issues and challenges of freelancing. AD and DM did statistics and data preparation and presentation. All authors contributed to the article and approved the submitted version.

## Conflict of interest

The authors declare that the research was conducted in the absence of any commercial or financial relationships that could be construed as a potential conflict of interest.

## Publisher’s note

All claims expressed in this article are solely those of the authors and do not necessarily represent those of their affiliated organizations, or those of the publisher, the editors and the reviewers. Any product that may be evaluated in this article, or claim that may be made by its manufacturer, is not guaranteed or endorsed by the publisher.
